# The effect of early postoperative acute pain on postoperative delirium in older persons undergoing abdominal surgery: a secondary analysis of multicenter prospective data

**DOI:** 10.1007/s41999-025-01367-w

**Published:** 2025-12-02

**Authors:** Hai-yue Ma, Xue-cai Lv, Chen Zhang, Zhuo-ning Zhang, Xin-yu Hao, Yan-hong Liu, Jiang-bei Cao, Wei-dong Mi, Li Tong, Qiang Fu

**Affiliations:** 1 The Department of Anaesthesiology, The First Medical Centre of Chinese People’s Liberation Army (PLA) General Hospital, Beijing, 100853 China; 2 Medical School of Chinese People’s Liberation Army (PLA) General Hospital, Beijing, 100853 China

**Keywords:** Delirium, Older persons, Postoperative acute pain, Abdominal surgery

## Abstract

**Purpose:**

Older persons frequently experience postoperative acute pain and delirium, however, there is little clinical data on their possible interrelationships.

**Methods:**

A secondary analysis of multicenter prospective data, including tertiary hospitals in Five Chinese Regions, from April 2020 to April 2022. Older persons (≥ 65 years) scheduled for elective abdominal surgery under general anesthesia, without auditory, visual, or cognitive impairments, who completed assessments for postoperative delirium, anxiety, and depression. The impact of early postoperative acute pain on postoperative delirium in older persons undergoing abdominal surgery was evaluated using univariate and multivariate logistic regression, propensity score matching, and subgroup analysis.

**Results:**

The study cohort included 2,674 patients. The median age was 70 years, and 66.5% of the patients were male. Delirium occurred in 13.2% (*n* = 354) of patients in postoperative 7 days (POD 7), with a significantly higher prevalence in the moderate-to-severe pain group (unadjusted: 16.3 vs. 9.6%, *p* < 0.001). Moderate-to-severe pain on POD1 showed a significant association with delirium occurrence in POD 7 according to univariate logistic regression (OR 1.83, 95% CI 1.445–2.319, *p* < 0.001), multivariable logistic regression (OR 1.63, 95% CI 1.274–2.084, *p* < 0.001), and propensity score matching (PSM) model (OR 1.44, 95% CI 1.108–1.879, *p* = 0.006).

**Conclusion:**

In older persons undergoing elective abdominal surgery, moderate-to-severe pain on POD 1 was significantly associated with both delirium and depressive symptoms within 7 days of surgery. Improved early postoperative analgesic strategies may aid in preventing delirium.

*Trial registration*: ClinicalTrials.gov identifier: NCT06964893 (2025-05-07).

**Supplementary Information:**

The online version contains supplementary material available at 10.1007/s41999-025-01367-w.

## Key summary points

**Aim:**

This study aimed to investigate the impact of moderate-to-severe acute pain on postoperative day 1 (POD1) on delirium in older persons undergoing abdominal surgery.

**Findings:**

In older persons undergoing elective abdominal surgery, moderate-to-severe pain on POD 1 was significantly associated with both delirium and depressive symptoms within 7 days of surgery.

**Message:**

Adequate management of early postoperative acute pain may help prevent postoperative delirium in older persons.

**Supplementary Information:**

The online version contains supplementary material available at 10.1007/s41999-025-01367-w.

## Introduction

Delirium, a prevalent and severe postoperative complication in older persons, is clinically characterized by attention deficits, impaired consciousness, and disturbances in orientation, memory, and perception [1]. The incidence of delirium following non-cardiac surgeries ranges from 13 to 50% [2], with abdominal surgery specifically associated with a 21% incidence rate [3]. Delirium leads to multiple adverse outcomes including prolonged hospitalization, diminished quality of life, persistent cognitive decline, increased postoperative morbidity, elevated healthcare expenditures, and a higher risk of mortality [4, 5]. Furthermore, postoperative negative emotional states such as anxiety and depression frequently occur in older surgical persons, significantly compromising clinical outcomes.

Postoperative pain management remains a significant clinical challenge despite advancements in surgical and anesthetic techniques. The incidence of moderate-to-severe acute postoperative pain persists at elevated levels, with studies reporting rates reaching 50% in some populations [6]. This is particularly concerning given the established association between pain and delirium in older surgical persons—a combination that frequently leads to adverse outcomes in the absence of timely prevention, diagnosis, and intervention. There is also emerging evidence of interconnections between pain, anxiety, and depression. Early postoperative acute pain is a modifiable and clinically significant indicator. Effective pain management strategies may therefore serve as preventive measures against delirium, anxiety, and depression in geriatric surgical patients.

Consequently, investigating the impact of early acute postoperative pain on delirium development and postoperative anxiety or depression symptoms holds significant clinical importance for optimizing peri-operative analgesia protocols and enhancing clinical outcomes in older surgical persons. This study will provide critical theoretical foundations for further optimizing comprehensive postoperative management protocols for older persons.

## Methods

### Ethical approval

This secondary analysis of multicenter prospective data involving human participants was in accordance with the ethical standards of the institutional and national research committee and with the 1964 Helsinki Declaration and its later amendments or comparable ethical standards. The Clinical Research Ethics Committee of the First Medical Center of the Chinese PLA General Hospital approved this study (No.: S2025-137-02).

## Study design and cohort

This multicenter secondary analysis of prospective data included older persons who underwent elective abdominal surgery between April 2020 and April 2022 across five provinces or municipalities in China (Guizhou, Nanjing, Hunan, Hubei, and Beijing). Clinical data were systematically collected for analysis.

The inclusion criteria were as follows: age ≥ 65 years; absence of auditory, visual, or cognitive impairments (operationally defined as a Chinese version of the Mini-Mental State Examination [MMSE] score ≤ 17); scheduled for elective abdominal surgery under general anesthesia with endotracheal intubation; complete assessments for postoperative delirium, anxiety, and depression. Patients were excluded if they met any of the following criteria: history of severe psychiatric disorders or chronic use of psychotropic medications (n = 715); NOTES: natural orifice transluminal endoscopic surgery (n = 454); vascular interventional procedures (n = 44); postoperative transfer to the intensive care unit (ICU) or death within 7 days after surgery (n = 1).

### Data collection

We collected baseline characteristics and demographic data of the patients, including clinical information in the following areas: (1) Baseline characteristics: age, sex, body mass index (BMI), American Society of Anesthesiologists (ASA) physical status score, smoking, alcohol consumption, preoperative history of chronic pain, and comorbidities (hypertension, diabetes mellitus, coronary artery disease, cerebrovascular disease, hepatic insufficiency, and renal insufficiency). (2) Pre-operative laboratory tests (the most recent prior to surgery): hemoglobin (Hb), white blood cell count, serum albumin. (3) Preoperative psychological assessments: anxiety and depression state evaluations. (4) Intra-operative variables: grade of operation, type of surgery, duration of surgery, transfusion, blood loss, intra-operative dexmedetomidine and nonsteroidal anti-inflammatory drugs (NSAIDs), drain, and patient-controlled intravenous analgesia (PCIA).

### Sample size

Statistical analyses were performed using PASS version 15.0. Based on an anticipated 4.7% intergroup difference in delirium incidence, with a two-sided α level of 0.05 and 80% power (β = 0.20), our preliminary sample size calculation indicated a minimum requirement of 733 patients per group[7]. The final cohort of 2,674 patients included in our study clearly exceeded this minimal threshold, ensuring adequate statistical power.

### Exposure

In this study, we defined the presence or absence of moderate-to-severe pain on POD1 as the exposure variable, assessed using the numerical rating scale (NRS) [8]. An NRS score < 4 on POD1 was classified as mild pain, while a score ≥ 4 indicated moderate-to-severe pain. Based on this exposure variable, patients were stratified into the mild pain group (NRS < 4) and moderate-to-severe pain group (NRS ≥ 4).

### Outcomes

The primary outcomes were the incidence of delirium and its subtypes during POD 7. Delirium was assessed at the bedside by trained staff using the Chinese version of the 3-Minute Diagnostic Interview for CAM-defined Delirium (3D-CAM) [9]. Assessments were conducted twice daily (in the morning and afternoon) throughout the postoperative period.

The secondary outcomes included the incidence and subtypes of anxiety or depression symptoms. Trained assessors evaluated postoperative anxiety using the Chinese version of the Generalized Anxiety Disorder-7 (GAD-7) scale, administered at the patient's bedside during the recovery period [10]. A score > 5 was defined as indicating anxiety symptoms, with scores of 5–8 indicating mild symptoms, 9–14 representing moderate anxiety, and 15–21 reflecting severe anxiety. Postoperative depressive symptoms were assessed by trained researchers using the validated Chinese version of the Patient Health Questionnaire-9 (PHQ-9) at the patient's bedside[11]. A score > 5 was defined as indicating depressive symptoms, with scores of 5–9 representing mild symptoms and 10–14 indicating moderate depression. A score of 15–27 was classified as moderate-to-severe depression. These psychological assessments were conducted once during the postoperative hospitalization period, scheduled between postoperative days 3–7.

For patients discharged before completing all assessments (delirium, anxiety, or depression), trained researchers performed standardized telephone follow-up evaluations using the same assessment protocols.

### Statistical analysis

We descriptively summarized patient characteristics stratified by the presence or absence of moderate-to-severe pain. Continuous variables were reported as means ± standard deviation for normally distributed data or median (IQR) for non-normally distributed data, with between-group comparisons performed using Student’s *t*-test or the Mann–Whitney *U* test, respectively. Categorical variables were summarized as frequencies (percentages) and analyzed using Pearson’s chi-squared test.

Univariate and multivariate regression analyses were performed to examine the relationship between acute pain and the incidence of early delirium. In the multivariate logistic regression model, a forced-entry method was applied, incorporating all predefined covariates. Variable selection for adjustment was guided by univariate screening and clinical plausibility regarding their association with delirium. To enhance the robustness of our findings, we performed PSM followed by subgroup analyses [12]. We adjusted for covariates exhibiting a standardized mean difference (SMD) > 0.2 between the mild and moderate-to-severe pain cohorts. Both the exposed and unexposed groups comprised 1072 participants each. Post-matching balance was confirmed by achieving an SMD < 0.2 for all included variables, indicating well-balanced baseline characteristics. Statistical significance was defined as a two-sided *P*-value of < 0.05. All statistical analyses were performed using the R software (version 4.0.1; R Foundation for Statistical Computing, Vienna, Austria).

## Results

**Fig. 1:**
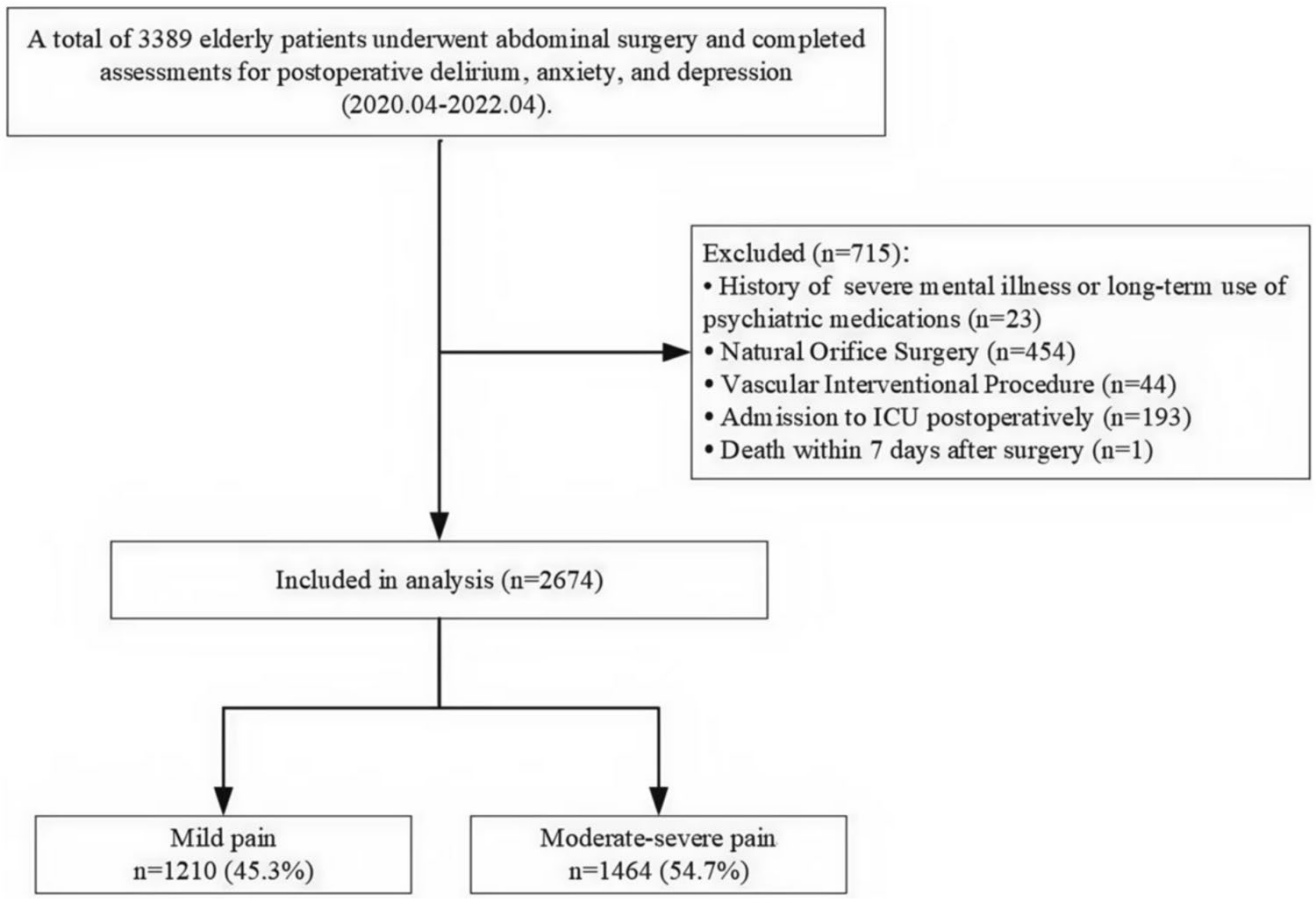
Analysis flow chart

We initially enrolled 3,389 patients aged 65 years or older. After excluding 715 patients based on the listed criteria, 2,674 were ultimately included in the analysis (Fig. 1).

The median age was 70 years (interquartile range [IQR]: 67–74), and 627 (23.4%) patients were aged ≥ 75 years. Notably, the patients (1,779; 66.5%) were male. The overall incidence rate of delirium in this cohort was 13.2%. Patients were stratified into mild pain and moderate-to-severe pain groups. Significant differences (*P* < 0.05) were observed between the groups in terms of ASA classification, surgical type, operative duration, drain, intra-operative dexmedetomidine, NSAIDs, and PCIA. Other variables showed no statistical differences. Compared with the mild pain group, a higher percentage of patients in the moderate-to-severe pain group had lower ASA classification, smoked, consumed alcohol, had low hemoglobin, preoperative anxiety, preoperative depression, open surgeries, prolonged operative time, drain, non-use of dexmedetomidine, NSAIDs, and PCIA (Table 1).

**Table 1:** Characteristics of the primary cohort before and after matching

Variable	Total	Before PSM (*n* = 2674)				After PSM (n = 2144)			
		Mild pain (n = 1210)	Moderate–severe pain (n = 1464)	*P*	SMD	Mild pain (n = 1072)	Moderate–severe pain (n = 1072)	*P*	SMD
*Preoperative variables*									
Age (median [interquartile range]), years	70.0 [67.0–74.0]	71.00 [67.0–74.0]	70.0 [67.0–74.0]	0.486	0.039	71.0 [68.0–74.0]	70.0 [67.0–74.0]	0.510	0.027
Male, *n* (%)	1779 (66.5)	808 (66.8)	971 (66.3)	0.805	0.010	696 (64.9)	724 (67.5)	0.201	0.056
BMI (median [interquartile range]), kg/m^2^	24.1 [22.1–25.7]	24.1 [22.3–25.7]	24.0 [22.0–25.7]	0.073	0.060	24.1 [22.3–25.8]	24.1 [22.0–25.6]	0.156	0.048
ASA*,* n* (%)				< 0.001	0.225			1.000	0.000
I/II	1899 (71.0)	795 (65.7)	1104 (75.4)			768 (71.6)	768 (71.6)		
III/V	775 (29.0)	415 (34.3)	360 (24.6)			304 (28.4)	304 (28.4)		
Comorbidity, *n* (%)				0.560	0.023			0.960	0.002
< 2	2034 (76.1)	914 (75.5)	1120 (76.5)			816 (76.1)	817 (76.2)		
≥ 2	640 (23.9)	296 (24.5)	344 (23.5)			256 (23.9)	255 (23.8)		
Smoke,* n* (%)	745 (27.9)	306 (25.3)	439 (30.0)	0.007	0.103	274 (25.6)	332 (31.0)	0.005	0.117
Alcohol, *n* (%)	710 (26.6)	297 (24.6)	413 (28.2)	0.033	0.081	271 (25.3)	309 (28.8)	0.065	0.078
Preoperative chronic pain, *n* (%)	88 (3.3)	48 (4.0)	40 (2.7)	0.075	0.076	44 (4.1)	22 (2.0)	0.006	0.145
Preoperative evaluation with scales									
Preoperative anxiety,* n* (%)	1025 (38.3)	452 (37.4)	573 (39.1)	0.345	0.037	392 (36.6)	427 (39.8)	0.120	0.067
Preoperative depression, *n* (%)	775 (29.0)	330 (27.3)	445 (30.4)	0.076	0.068	289 (27.0)	330 (30.8)	0.051	0.083
*Preoperative laboratory examinations*									
Hemoglobin, g dl^−1^,* n* (%)				0.018	0.092			0.318	0.043
< 13.0	1470 (55.0)	635 (52.5)	835 (57.0)			575 (53.6)	598 (55.8)		
≥ 13.0	1204 (45.0)	575 (47.5)	629 (43.0)			497 (46.4)	474 (44.2)		
Albumin (median [interquartile range]), g/L	39.7 [37.1–41.9]	39.7 [37.2–41.8]	39.7 [37.0–42.0]	0.960	0.006	39.9 [37.0–41.9]	40.0 [37.2–42.1]	0.517	0.024
WBC count (median [interquartile range]), mmol/L	5.7 [4.7–6.8]	5.7 [4.6–6.8]	5.7 [4.7–6.8]	0.778	0.012	5.7 [4.6–6.7]	5.8 [4.7–6.8]	0.354	0.031
*Surgical/anesthetic parameters*									
Grade of operation, *n* (%)				0.494	0.027			0.965	0.002
< 3	1020 (38.1)	453 (37.4)	567 (38.7)			429 (40.0)	428 (40.0)		
≥ 3	1654 (61.9)	757 (62.6)	897 (61.3)			643 (60.0)	644 (60.1)		
Type of surgical*, *n* (%)				< 0.001	0.221			0.043	0.092
Minimally invasive	2210 (82.6)	1060 (87.6)	1150 (78.6)			922 (86.0)	953 (89.0)		
Open	464 (17.4)	150 (12.4)	314 (21.4)			150 (14.0)	119 (11.1)		
Duration of surgery, min, *n* (%)				0.003	0.114			0.062	0.080
< 180	1439 (53.8)	689 (57.0)	750 (51.2)			618 (57.7)	575 (53.6)		
≥ 180	1235 (46.2)	521 (43.0)	714 (48.8)			454 (42.4)	497 (46.4)		
Blood loss (median [interquartile range]), ml	100.0 [50.0–116.0]	100.0 [50.0–116.0]	100.0 [50.0–116.0]	0.555	0.013	100.0 [30.0–116.0]	100.0 [50.0–116.0]	0.387	0.019
Blood transfusion,* n* (%)	240 (9.0)	117 (9.7)	123 (8.4)	0.256	0.045	103 (9.6)	103 (9.6)	1.000	0.000
Intraoperative medication									
Dexmedetomidine*,* n* (%)	659 (24.6)	355 (29.3)	304 (20.8)	< 0.001	0.211	244 (22.8)	225 (21.0)	0.321	0.044
NSAIDs,* n* (%)	1420 (53.1)	609 (50.3)	811 (55.4)	0.009	0.102	567 (52.9)	581 (54.2)	0.544	0.026
PCIA*, *n* (%)	1326 (49.6)	498 (41.2)	828 (56.6)	< 0.001	0.311	498 (46.5)	533 (49.2)	0.130	0.065
Drain, *n* (%)	2292 (85.7)	1011 (83.6)	1281 (87.5)	0.004	0.119	887 (82.7)	918 (85.6)	0.067	0.082

The data are shown as the median [interquartile range], *n* (%)

Abbreviations: SMD, standardized mean difference; PSM, propensity score matching; BMI, body mass index; ASA, American Society of Anesthesiologists Physical Status Classification System; WBC, white blood cell; NSAIDs, non-steroidal anti-inflammatory drugs; PCIA, patient—controlled intravenous analgesia

^*^Variables included in the Propensity score

We used four logistic regression models to analyze the association between moderate-to-severe acute postoperative pain and delirium on POD 1 in older persons. In the univariate analysis, moderate-to-severe acute pain was significantly associated with an increased risk of delirium (OR = 1.83, 95% CI 1.445 to 2.319, *P* < 0.001). The univariate logistic regression results are presented in Supplementary Table 1.

In all multivariable regression models, moderate-to-severe acute pain remained an independent risk factor for delirium: Model 2 OR = 1.82 (95% CI 1.434 to 2.317, *P* < 0.001); Model 3 OR = 1.61 (95% CI 1.266 to 2.055, *P* < 0.001); Model 4 OR = 1.63 (95% CI 1.274 to 2.084, *P* < 0.001); Model PSM OR = 1.44 (95% CI 1.108 to 1.879, *P* = 0.006) (Table 2). The detailed specifications of each model are provided in Supplementary Table 2.

**Table 2:** Association between postoperative day 1: moderate-severe acute pain and delirium with logistic regression models and propensity score matching (PSM) analysis

	OR	95% CI	*P*
Model 1^a^	1.83	1.445–2.319	< 0.001
Model 2^b^	1.82	1.434–2.317	< 0.001
Model 3^c^	1.61	1.266–2.055	< 0.001
Model 4^d^	1.63	1.274–2.084	< 0.001
Model PSM (n = 2144)	1.44	1.108–1.879	0.006

^a^Model 1 a univariate logistic regression analysis

^b^Model 2 adjusted for age, gender, body mass index (BMI), American Society of Anesthesiologists (ASA) physical status score, smoking, alcohol consumption, preoperative history of chronic pain, and comorbidity, hemoglobin (Hb), white blood cell count, serum albumin, preoperative psychological assessments: anxiety and depression state evaluations

^c^Model 3 adjusted for grade of operation, type of surgery, duration of surgery, transfusion, blood loss, intraoperative dexmedetomidine, nonsteroidal anti-inflammatory drugs (NSAIDs), drain, patient-controlled intravenous analgesia (PCIA)

^d^Model 4 adjusted for model 2 plus model 3

^e^Model PSM a multivariable logistic regression

**Fig. 2:**
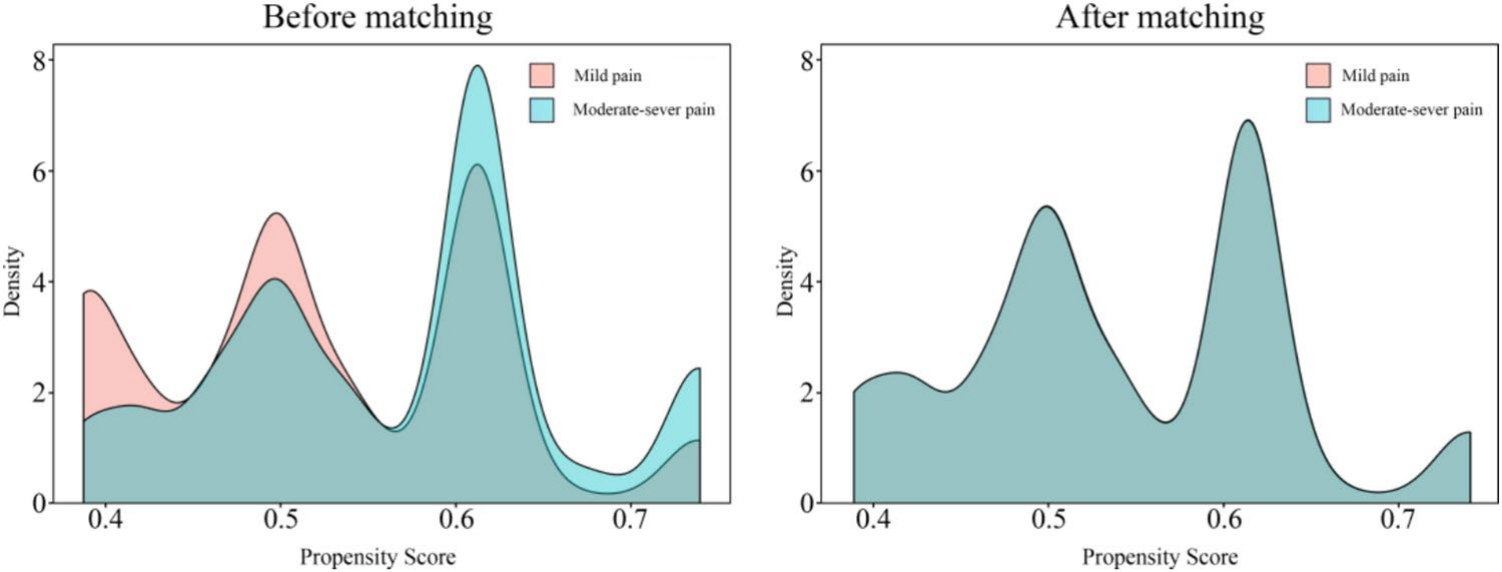
Distribution of propensity scores before and after matching between the mild pain and moderate-severe pain groups

PSM was performed at a 1:1 ratio to balance the baseline covariates. Variables with an SMD > 0.2 (ASA classification, surgical type, intra-operative dexmedetomidine, PCIA) were matched between the mild and moderate-to-severe pain groups. Kernel density plots illustrated the propensity score (PS) distributions before and after matching (Fig. 2). Post-matching, each group comprised 1072 patients, with all baseline characteristics balanced (SMD < 0.2; Table 1).

Within the first 7 postoperative days, the incidence of delirium was significantly higher in the moderate-to-severe pain group compared to the mild pain group (16.3 vs. 9.6%, *p* < 0.001). Among delirium subtypes, significant differences were observed in hypoactive delirium (10.1 vs. 6.1%). After 1:1 PSM (n = 2,144), the association between moderate-to-severe pain and delirium remained significant (15.0 vs. 10.4%, *p* = 0.002), with persistent significant differences in hypoactive delirium between the groups (*p* = 0.025) (Table 3).

**Table 3:** Primary and secondary outcomes before and after propensity score matching (PSM)

Characteristics	Before PSM ( n = 2674)			After PSM ( n = 2144)		
	Mild pain (n = 1210)	Moderate–severe pain (n = 1464)	*P*	Mild pain (n = 1072)	Moderate–severe pain (n = 1072)	*P*
*Primary outcomes*, (*n*%)						
Postoperative delirium	116(9.6%)	238(16.3%)	< 0.001	112(10.4%)	161(15.0%)	0.002
Hypoactive delirium	74(6.1%)	148(10.1%)	< 0.001	71(8.5%)	99(9.2%)	0.025
Hyperactive delirium	14(1.2%)	29(2.0%)	0.092	13(0.2%)	21(2.0%)	0.167
Mixed delirium	28(2.3%)	61(4.2%)	0.008	28(1.7%)	41(3.8%)	0.112
*Secondary outcomes*, (*n*%)						
Postoperative anxiety	336(27.8%)	410(28.0%)	0.892	295(27.5%)	290(27.0%)	0.808
Mild anxiety	205(16.9%)	267(18.2%)	0.366	182(17.0%)	183(17.1%)	0.954
Moderate anxiety	96(7.9%)	110(7.5%)	0.685	78(7.3%)	86(8.0%)	0.516
Severe anxiety	35(2.9%)	33(2.3%)	0.297	35(3.3%)	21(2.0%)	0.058
Postoperative depression	247(20.4%)	389(26.6%)	< 0.001	224(20.9%)	290(27.0%)	0.001
Mild depression	138(11.4%)	200(13.7%)	0.081	127(11.8%)	139(13.0%)	0.432
Moderate depression	78(6.4%)	132(9.0%)	0.014	67(6.2%)	109(10.2%)	0.001
Moderate–severe depression	31(2.6%)	57(3.9%)	0.055	30(2.8%)	42(3.9%)	0.150

Regarding secondary outcomes, before matching, the moderate-to-severe pain group showed a higher incidence of depressive symptoms (26.6 vs. 20.4%, *p* < 0.001), with the most pronounced difference in moderate depressive symptoms (9.0 vs. 6.4%, *p* = 0.014). After matching, significant differences persisted in overall depressive symptoms (27.0 vs. 20.9%, *p* = 0.001) and moderate depressive symptoms (10.2 vs. 6.2%, *p* = 0.001). However, no significant association was found between early postoperative acute pain and the incidence of overall postoperative anxiety symptoms or different anxiety levels, either before or after matching (Table 3). Results of the multivariate logistic regression for secondary outcomes are presented in Supplementary Table 3.

**Fig. 3:**
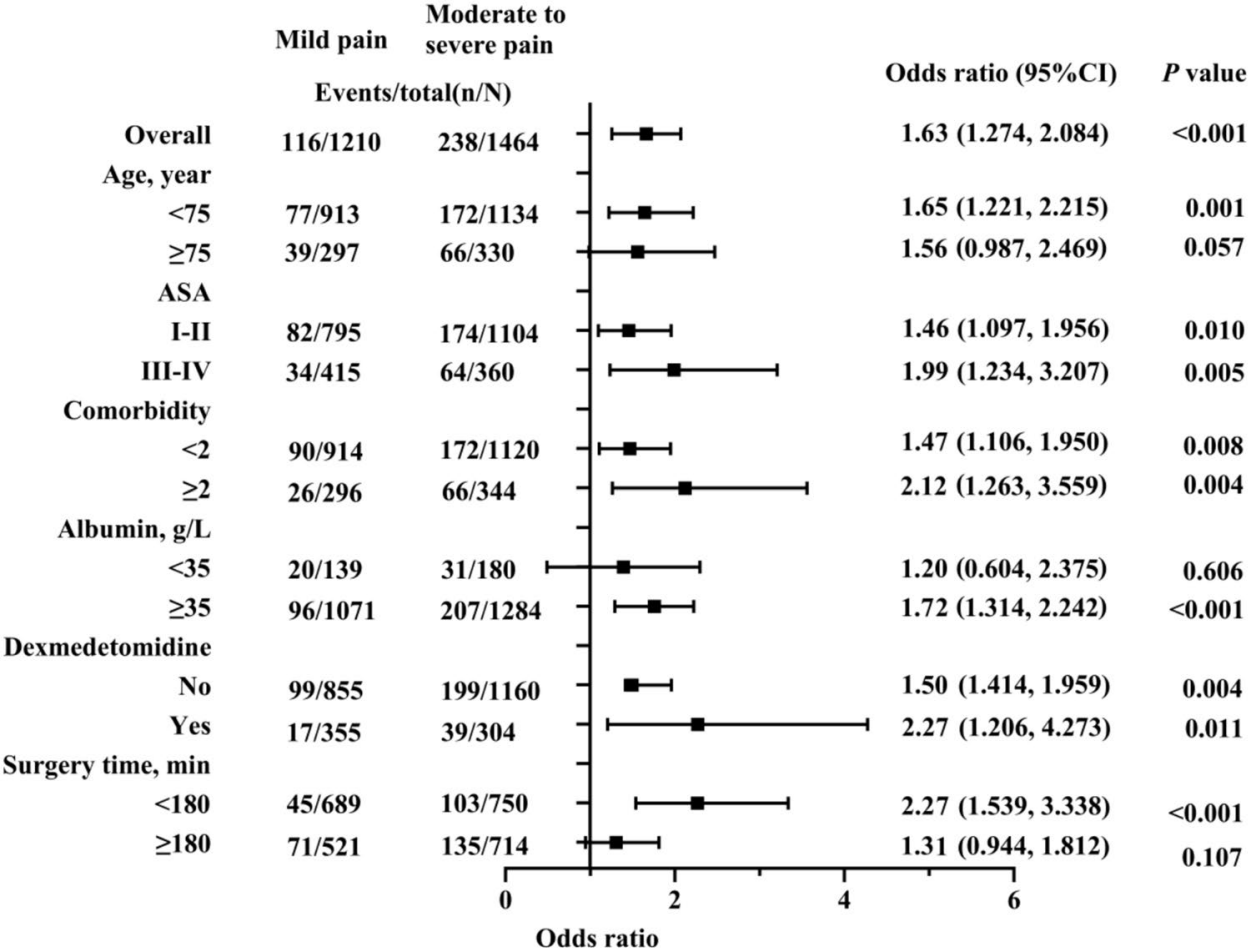
Subgroup analysis of the association between postoperative moderate-to-severe acute pain and POD, stratified by age, ASA classification, comorbidity, albumin levels, intra-operative dexmedetomidine use, and surgical duration. OR, odds ratio; CI, confidence interval; ASA, American Society of Anesthesiologists classification

We performed subgroup analyses in 2674 older persons undergoing abdominal surgery, stratified by age, ASA classification, comorbidities, intra-operative dexmedetomidine, preoperative albumin levels, and operative duration. Significant associations between moderate-to-severe acute postoperative pain and delirium were observed in the following subgroups: ASA classification, comorbidity, intra-operative dexmedetomidine use, age < 75 years, albumin ≥ 35 g/L, and operative duration < 180 min (Fig. 3).

## Discussion

This secondary analysis of multicenter prospective data included 2674 older persons undergoing elective abdominal surgery. Key findings include that the incidence of moderate-to-severe pain during POD 1 was 54.7%, with this group demonstrating a significantly higher incidence of delirium than the mild pain group. Univariate analysis, multifactorial logistic regression, and PSM analyses consistently demonstrated a significant association between moderate-to-severe acute postoperative pain and the development of delirium as well as postoperative depressive states, but no significant link with postoperative anxiety states. Both before and after PSM, moderate-to-severe acute postoperative pain showed significant associations with hypoactive delirium and moderate postoperative depression.

The incidence of delirium in older persons varies by surgical type. Available data indicate notable differences in delirium risk across procedures: cardiac surgery patients show the highest rate (26%) [13], resulting in an overall lower rate of 18.4% in non-cardiac surgical patients [14]. A prospective observational study of 184 thoracic and abdominal surgery cases reported a delirium incidence of 14.6% [15]. This finding aligns closely with the overall delirium incidence observed in this study (13.2%) among older persons undergoing abdominal surgery.

The occurrence of delirium is influenced by multiple factors, with the risk significantly associated with patient-specific characteristics. A meta-analysis encompassing 21 studies found that age, BMI, ASA classification, comorbidities, and preoperative C-reactive protein levels were strongly correlated with delirium risk [16]. In addition to patient-related factors, intra-operative variables also contribute to delirium development. In a prospective cohort of geriatric patients undergoing major abdominal procedures, open laparotomy, intra-operative transfusion, and a diagnosis of colorectal cancer were independently associated with an increased incidence of postoperative delirium [17].

Clinical studies have further supported a link between postoperative pain and delirium. A meta-analysis of 30 studies demonstrated a significant association between pain and delirium [18]. The present study aligns with these findings, confirming a significant correlation between moderate-to-severe acute postoperative pain and delirium development in older persons undergoing elective abdominal surgery.

In spite of these known risk associations, the incidence of acute postoperative pain remains high. Available data show that even in less invasive ambulatory surgeries, the incidence of moderate-to-severe pain within 24 h post-surgery remains as high as 30% [19]. By contrast, the incidence of acute postoperative pain in comprehensive surgery was 48% [20] while for patients undergoing abdominal surgery, it reached 45.6% [21]. A 2016 cross-sectional observational study by British scholars, involving over 15,000 surgical patients, revealed that the prevalence of moderate postoperative pain in non-obstetric surgeries was 37.2%, with severe pain occurring in 11% of cases [22]. In the Chinese population, the prevalence of moderate-to-severe postoperative pain was 48.7%, with the incidence of acute postoperative pain following abdominal surgery exceeding 50% [23]. Notably, inadequate analgesic measures not only directly impede patient recovery but may also be a significant predisposing factor for delirium.

The mechanism by which postoperative pain contributes to delirium involves a complex pathophysiological process with multiple interacting factors and pathways. Surgical trauma-induced tissue damage activates the innate immune system, triggering the release of pro-inflammatory cytokines such as IL-1β, IL-6, and TNF-α. These inflammatory mediators not only enhance peripheral and central sensitization, leading to nociceptive hypersensitivity, but also impair cognitive function by disrupting the blood–brain barrier and altering neurotransmitter homeostasis. Additionally, pain may activate the hypothalamic–pituitary–adrenal (HPA) axis, stimulating increased glucocorticoid secretion [24]. Prolonged elevation of cortisol levels can cause hippocampal neuronal damage, negatively affecting memory and cognitive functions. Pain also disrupts neurotransmitter systems, promoting delirium by interfering with the normal metabolism of acetylcholine, dopamine, and serotonin [25]. Furthermore, painful stimuli increase the production of reactive oxygen species (ROS) in the hippocampus, leading to oxidative neuronal damage, which also increases the risk of delirium [26, 27].

Peri-operative anxiety and depression are common negative emotional states associated with poor clinical outcomes. Older persons frequently experience mood disorders such as anxiety and depression postoperatively. Existing studies demonstrate a bidirectional relationship between negative emotions and acute pain, as depression and anxiety lower pain thresholds and amplify pain perception, while persistent nociceptive stimuli exacerbate mood disorders, creating a vicious cycle [28]. The multivariate analysis of secondary outcomes in this study revealed a significant association between moderate-to-severe acute postoperative pain and the onset of depressive symptoms. Analysis of Taiwan’s National Health Insurance Research Database (N = 141,466) identified chronic postsurgical pain as an independent predictor of postoperative depressive symptoms in patients aged > 20 years undergoing major surgery [29]. Further supporting this correlation, a multicenter prospective cohort study reported that patients with severe acute postpartum pain faced a threefold higher risk of postnatal depression compared to those with mild pain [30]. Our results corroborate these observations, confirming that moderate-to-severe acute postoperative pain is significantly correlated with subsequent depressive states.

Several studies have demonstrated that peri-operative dexmedetomidine significantly reduces the incidence of delirium in older persons undergoing noncardiac surgery [31, 32]. However, our subgroup analysis revealed a significant association between acute postoperative pain and delirium occurrence, regardless of dexmedetomidine use. This suggests that pain may contribute to delirium development through pathways independent of dexmedetomidine’s mechanism of action, highlighting the need for a multimodal peri-operative management strategy—incorporating both delirium-preventing medications (e.g., dexmedetomidine) and optimized pain control. Frailty is a clinical state characterized by decreased physiological reserves and increased vulnerability to stressors. It is highly prevalent among older surgical patients and is closely associated with delirium [33]. Advanced age and hypoalbuminemia are both well-established important risk factors for frailty [34]. In line with this, subgroup analyses in our study revealed heterogeneity in the association between acute postoperative pain and delirium: no significant association was observed between moderate-to-severe acute pain and postoperative delirium in patients aged ≥ 75 years, with albumin levels < 35 g/L, or with an operative duration ≥ 180 min. We hypothesize that this phenomenon stems from the severe depletion of physiological reserves and the consequent high risk of frailty commonly present in these patient subgroups. In such physiologically vulnerable patients, the pathway to delirium may be primarily driven by their underlying physiological fragility, thereby attenuating the relative contribution of single acute stressors such as pain. Therefore, for these high-risk populations, clinical intervention should extend beyond pain management alone and instead adopt a multidimensional strategy that integrates pain control with the optimization of nutritional, cognitive, functional, and medication status. Future studies utilizing larger cohorts or incorporating biomarker stratification are warranted to further elucidate the intrinsic mechanisms underlying these subgroup differences.

In spite of its valuable findings, our study has several limitations. Firstly, while we employed robust statistical methods including PSM and univariate or multivariate logistic regression to control for confounding factors, potential residual confounders, including unmeasured or unforeseen variables, may affect the pain–delirium association. Secondly, the lack of ward analgesia documentation (including opioid or non-opioid use) constitutes a major limitation, potentially introducing unmeasured confounding. Thirdly, a key limitation is the absence of data on important geriatric variables, due to both the exclusion of patients with specific conditions (such as preexisting mental health disorders, sensory or cognitive impairments, or postoperative ICU admission) and the lack of systematic data collection for other parameters, including incontinence, nutritional status, frailty, and activities of daily living. These limitations likely resulted in unmeasured confounding, potentially affecting the accuracy of the estimated relationship between acute postoperative pain and delirium. Future prospective studies should integrate a comprehensive geriatric assessment to better delineate the independent role of pain within delirium’s multifactorial etiology.

## Conclusions

In our cohort of older persons undergoing elective abdominal surgery, moderate-to-severe acute pain on POD1 was significantly associated with both the development of delirium and depressive symptoms during POD 7. These findings highlight the need to optimize postoperative analgesia to mitigate neuropsychiatric complications in geriatric surgical populations. Future randomized controlled trials should investigate targeted pain management strategies to reduce the incidence of delirium in this vulnerable population.

## Supplementary Information

Below is the link to the electronic supplementary material.

Supplementary file1 (DOCX 36 KB)

## Data Availability

The datasets generated during and/or analyzed during the current study are available from the corresponding author on reasonable request.
