# Supplementary material for: The effect of early postoperative acute pain on postoperative delirium in older persons undergoing abdominal surgery: a secondary analysis of multicenter prospective data
Source: Eur Geriatr Med. 2025 Dec 2;17(2):851–61. doi: 10.1007/s41999-025-01367-w (PMC13109186; doi:10.1007/s41999-025-01367-w)
Supplement: Supplementary file 1 — Supplementary file1 (DOCX 36 KB) [file 41999_2025_1367_MOESM1_ESM.docx]

**Table S1** Multivariable logistic regression analyses for delirium in the propensity score matching ( PSM ) model

|  | OR | 95% CI | *P* |
| --- | --- | --- | --- |
| Age,years* | 1.05 | 1.024-1.066 | <0.001 |
| Male, n (%) | 1.38 | 1.093-1.730 | 0.007 |
| BMI,kg/m²* | 0.98 | 0.942-1.013 | 0.208 |
| ASA, n (%) | 0.93 | 0.724-1.192 | 0.563 |
| Comorbidity, n (%) | 1.14 | 0.879-1.467 | 0.331 |
| Smoke, n (%) | 0.85 | 0.659-1.101 | 0.221 |
| Alcohol, n (%) | 0.90 | 0.698-1.169 | 0.439 |
| Perioperative chronic pain, n (%) | 1.04 | 0.558-1.925 | 0.911 |
| Preoperative laboratory examinations |  |  |  |
| Haemoglobin, n (%) | 0.76 | 0.606-0.957 | 0.020 |
| WBC count, 109/L* | 1.00 | 0.948-1.057 | 0.973 |
| Albumin, g/L* | 0.97 | 0.943-0.996 | 0.023 |
| Preoperative evaluation with scales |  |  |  |
| Preoperative anxiety, n (%) | 1.17 | 0.929-1.464 | 0.185 |
| Preoperative depression, n (%) | 1.36 | 1.075-1.726 | 0.011 |
| Surgical-related information |  |  |  |
| Grade of operation, n (%) | 1.13 | 0.899-1.432 | 0.289 |
| Type of surgical, n (%) | 1.39 | 1.055-1.828 | 0.019 |
| Duration of surgery, n (%) | 1.75 | 1.392-2.190 | <0.001 |
| Blood loss, ml* | 1.00 | 1.000-1.001 | 0.032 |
| Blood transfusion, n (%) | 0.89 | 0.595-1.338 | 0.580 |
| Intraoperative medication |  |  |  |
| Dexmedetomidine, n (%) | 0.54 | 0.396-0.722 | <0.001 |
| NSAIDs, n (%) | 1.11 | 0.887-1.391 | 0.360 |
| PCIA, n (%) | 1.85 | 1.471-2.332 | <0.001 |
| Drain, n (%) | 2.64 | 1.701-4.080 | <0.001 |

* indicate continuous variables

BMI, body mass index; ASA, American Society of Anesthesiologists Physical status

classification system; WBC, white blood cell; NSAIDs, non-steroidal anti-inflammatory drugs; PCIA, patient-controlled intravenous analgesia.

**Table S2** Detailed results of the multivariable logistic regression analyses for Model 2, 3, 4 and propensity score matching ( PSM )

|  | OR | 95% CI | *P* |
| --- | --- | --- | --- |
| **Model 2** |  |  |  |
| Postoperative day 1: moderate-severe acute pain | 1.82 | 1.434-2.317 | <0.001 |
| Age,years* | 1.04 | 1.023-1.067 | <0.001 |
| Male,n(%) | 1.33 | 1.007-1.756 | 0.044 |
| BMI,kg/m²* | 0.99 | 0.957-1.032 | 0.742 |
| ASA,n(%) | 0.92 | 0.711-1.192 | 0.531 |
| Comorbidity,n(%) | 1.12 | 0.859-1.445 | 0.414 |
| Smoke,n(%) | 0.97 | 0.709-1.325 | 0.844 |
| Alcohol,n(%) | 1.09 | 0.799-1.490 | 0.585 |
| Perioperative chronic pain, n (%) | 0.96 | 0.506-1.811 | 0.892 |
| Preoperative laboratory examinations |  |  |  |
| Haemoglobin, n (%) | 0.97 | 0.746-1.269 | 0.841 |
| WBC count, 109/L* | 1.00 | 0.950-1.058 | 0.923 |
| Albumin, g/L* | 0.98 | 0.947-1.006 | 0.112 |
| Preoperative evaluation with scales |  |  |  |
| Preoperative anxiety, n (%) | 1.03 | 0.795-1.337 | 0.817 |
| Preoperative depression, n (%) | 1.28 | 0.973-1.685 | 0.077 |
| **Model 3** |  |  |  |
| Postoperative day 1: moderate-severe acute pain | 1.61 | 1.266-2.055 | <0.001 |
| Surgical-related information |  |  |  |
| Grade of operation, n (%) | 0.91 | 0.714-1.717 | 0.477 |
| Type of surgical, n (%) | 1.12 | 0.837-1.491 | 0.453 |
| Duration of surgery, n (%) | 1.50 | 1.178-1.921 | 0.001 |
| Blood loss, ml* | 0.30 | 1.000-1.001 | 0.295 |
| Blood transfusion, n (%) | 0.97 | 0.643-1.470 | 0.894 |
| Intraoperative medication |  |  |  |
| Dexmedetomidine, n (%) | 0.66 | 0.472-0.921 | 0.015 |
| NSAIDs, n (%) | 0.86 | 0.668-1.118 | 0.266 |
| PCIA, n (%) | 1.42 | 1.061-1.093 | 0.018 |
| Drain, n (%) | 1.93 | 1.218-3.061 | 0.005 |
| **Model 4** |  |  |  |
| Postoperative day 1: moderate-severe acute pain | 1.63 | 1.274-2.084 | <0.001 |
| Age,years* | 1.06 | 1.032-1.078 | <0.001 |
| Male,n (%) | 1.36 | 1.019-1.802 | 0.036 |
| BMI,kg/m²* | 0.99 | 0.950-1.026 | 0.510 |
| ASA,n (%) | 1.02 | 0.771-1.345 | 0.896 |
| Comorbidity,n (%) | 1.14 | 0.878-1.488 | 0.322 |
| Smoke,n (%) | 0.93 | 0.683-1.278 | 0.670 |
| Alcohol,n (%) | 1.01 | 0.735-1.381 | 0.962 |
| Perioperative chronic pain, n (%) | 0.95 | 0.495-1.820 | 0.874 |
| Preoperative laboratory examinations |  |  |  |
| Haemoglobin, n (%) | 1.00 | 0.764-1.315 | 0.984 |
| WBC count, 109/L* | 1.01 | 0.953-1.068 | 0.763 |
| Albumin, g/L* | 0.98 | 0.948-1.010 | 0.175 |
| Preoperative evaluation with scales |  |  |  |
| Preoperative anxiety, n (%) | 1.09 | 0.837-1.423 | 0.520 |
| Preoperative depression, n (%) | 1.25 | 0.943-1.648 | 0.122 |
| Surgical-related information |  |  |  |
| Grade of operation, n (%) | 0.94 | 0.724-1.206 | 0.604 |
| Type of surgical, n (%) | 1.05 | 0.777-1.410 | 0.763 |
| Duration of surgery, n (%) | 1.57 | 1.224-2.017 | <0.001 |
| Blood loss, ml* | 1.00 | 1.000-1.001 | 0.254 |
| Blood transfusion, n (%) | 1.05 | 0.688-1.588 | 0.835 |
| Intraoperative medication |  |  |  |
| Dexmedetomidine, n (%) | 0.66 | 0.466-0.927 | 0.017 |
| NSAIDs, n (%) | 0.91 | 0.704-1.187 | 0.501 |
| PCIA, n (%) | 1.54 | 1.138-2.080 | 0.005 |
| Drain, n (%) | 2.02 | 1.269-3.220 | 0.003 |
| **Model PSM** |  |  |  |
| Postoperative day 1: moderate-severe acute pain | 1.44 | 1.108-1.8779 | 0.006 |
| Age,years* | 1.04 | 1.014-1.065 | 0.002 |
| Male,n (%) | 1.27 | 0.917-1.759 | 0.150 |
| BMI,kg/m²* | 0.99 | 0.952-1.037 | 0.755 |
| ASA,n (%) | 1.11 | 0.822-1.500 | 0.495 |
| Comorbidity,n (%) | 1.21 | 0.901-1.626 | 0.204 |
| Smoke,n (%) | 0.99 | 0.697-1.403 | 0.949 |
| Alcohol,n (%) | 0.97 | 0.68-1.385 | 0.869 |
| Perioperative chronic pain, n (%) | 0.43 | 0.149-1.207 | 0.108 |
| Preoperative laboratory examinations |  |  |  |
| Haemoglobin, n (%) | 0.93 | 0.682-1.262 | 0.634 |
| WBC count, 109/L* | 1.01 | 0.95-1.078 | 0.719 |
| Albumin, g/L* | 0.98 | 0.947-1.016 | 0.286 |
| Preoperative evaluation with scales |  |  |  |
| Preoperative anxiety, n (%) | 1.08 | 0.796-1.454 | 0.636 |
| Preoperative depression, n (%) | 1.27 | 0.922-1.737 | 0.145 |
| Surgical-related information |  |  |  |
| Grade of operation, n (%) | 0.96 | 0.717-1.280 | 0.770 |
| Type of surgical, n (%) | 0.94 | 0.631-1.389 | 0.743 |
| Duration of surgery, n (%) | 1.61 | 1.215-2.136 | 0.001 |
| Blood loss, ml* | 1.00 | 0.999-1.001 | 0.672 |
| Blood transfusion, n (%) | 1.12 | 0.725-1.735 | 0.607 |
| Intraoperative medication |  |  |  |
| Dexmedetomidine, n (%) | 0.74 | 0.509-1.073 | 0.111 |
| NSAIDs, n (%) | 0.91 | 0.678-1.227 | 0.542 |
| PCIA, n (%) | 1.43 | 1.034-1.982 | 0.031 |
| Drain, n (%) | 2.02 | 1.222-3.334 | 0.006 |

BMI, body mass index; ASA, American Society of Anesthesiologists Physical status

classification system; WBC, white blood cell; NSAIDs, non-steroidal anti-inflammatory drugs; PCIA, patient-controlled intravenous analgesia.

**Table S3** Multivariable analysis of secondary outcomes

|  | Overall | Before PSM (n = 2674) | | Overall | After PSM (n=2144) | |
| --- | --- | --- | --- | --- | --- | --- |
|  |  | Adjusted OR (95%CI) | Adjusted P |  | Adjusted OR (95%CI) | Adjusted P |
| Postoperative anxiety | 746(27.9%) | 0.98 (0.800-1.210) | 0.874 | 585(27.3%) | 0.94(0.747-1.175) | 0.574 |
| Postoperative depression | 636(23.8%) | 1.35 (1.103-1.663) | 0.004 | 514(24.0%) | 1.36(1.090-1.701) | 0.007 |

PSM propensity score matching.
